# Dyadic Successful Aging and the Limits of Agency

**DOI:** 10.1093/geroni/igab046.2784

**Published:** 2021-12-17

**Authors:** Markus Klingel

**Affiliations:** TU Dortmund University, Germany), Dortmund, Nordrhein-Westfalen, Germany

## Abstract

With increasing life expectancy, late life has become a longer, crucial part of the individual and dyadic life course. New opportunities, tasks and decisions emerged. Successful aging norms emphasize agency and autonomy. This can be activating, but also alienating. With increasing constraints, agency is limited and ideals of autonomy become dysfunctional. This challenges also relationships. Aging, functional losses and approaching death threaten dyadic satisfaction and functionality. Potentially, successful aging norms could erode dyadic solidarity when needed the most: in late life. This mixed-methods longitudinal study combines interviews and questionnaires at three observations across five years. Its focus lies on change over time and findings at observation three. The sample consists of eight German couples (78-86 years old, 50-65 years married, high relationship satisfaction, white, urban). What does aging mean for individualized actors? How do aging couples negotiate, decide and act on aging, autonomy and death? How do successful aging norms modulate dyadic aging? Overall, actors have internalized successful aging and benefit by influencing their health positively. However, this has become ambivalent. Actors increasingly perceive their future as limited and beyond individual control. Acceptance of losses that challenge the self is difficult, autonomy ideals burdensome and death salient. As individual agency is constrained, the dyad is still a functional stronghold against aging. Yet, it has to adapt as well to – potentially differential - individual aging. Losses can and do threaten couples’ functional and emotional unity. Four patterns of self-dyad dynamics emerged and exemplify tensions between individualized and dyadic successful aging.

